# Structural Behavior of RC Beams Containing Unreinforced Drilled Openings with and without CFRP Strengthening

**DOI:** 10.3390/polym14102034

**Published:** 2022-05-16

**Authors:** Alaa A. El-Sisi, Hesham M. El-Emam, Abd El-Monem I. El-Kholy, Seleem S. Ahmad, Hossam M. Sallam, Hani A. Salim

**Affiliations:** 1 Civil and Environmental Engineering, University of Missouri, Columbia, MO 65211, USA; salimh@missouri.edu; 2 Structural Engineering, Zagazig University, Zagazig 44511, Egypt; 3 Material Engineering, Zagazig University, Zagazig 44511, Egypt; hmalemam@eng.zu.edu.eg (H.M.E.-E.); ssdawod@eng.zu.edu.eg (S.S.A.); hem_sallam@zu.edu.eg (H.M.S.)

**Keywords:** strengthening, drilled opening, CFRP, finite element, concrete beam, blast hazard, ANSYS

## Abstract

Construction deficiencies can cause serious problems that significantly decrease the design strength of concrete structures, such as the unreinforced drilled openings. With the absence of sufficient reinforcement, the stress concentrations generated around the opening corners produce cracks in the beams. The size and location of the opening significantly affect the behavior of the beam under static and dynamic load. In this work, an experimental and numerical program was performed to investigate the behavior of drilled reinforced concrete beams with and without strengthening using CFRP sheets. Energy absorption and SDOF analyses were performed to preliminary assess the behavior of the beams under the dynamic load, such as blast. One control beam without any openings, six beams with tension-zone openings, and six beams with shear-zone openings were tested. It was found that the samples with tension-zone openings were slightly affected by the opening, where the reduction in the ultimate strength was approximately 7 to 14%. The beams were able to recover up to 46% of the lost strength by CFRP strengthening. On the other hand, the shear-zone opening significantly decreased the strength and energy absorption and increased the blast response. It can be concluded that it is not recommended to drill any opening at the shear zone as strength loss can reach 57% even with the strengthening, especially for blast resisting structure; in addition, the strength recovered from approximately 11.95 to 32.46% only. The finite element model was able to predict the strength of the beams. The results were closer in the case of tension-zone opening than those in the case of the shear-zone opening. Shear cracks were observed at the corners of the openings even if the opening exists at the tension zone. A reduction in the density of cracks can be observed after the strengthening, where the FRP sheet decreases the stress in the concrete.

## 1. Introduction

Design codes require a special reinforcement around the openings, which is very difficult to be achieved in the case of after construction or drilled openings. These openings might be needed to enable many facilities such as the passage of ducts and pipes for the heating, air-conditioning, sewage, water supply systems, electricity, telephone, and internet cables through the beams. In the absence of qualified and experienced engineering teams, these openings might be drilled in the wrong places, causing high values of stress concentrations. Stress concentration can cause severe cracks around the unreinforced drilled opening, which reduces the load-carrying capacity and stiffness and increases the deflection and crack widths in the beam.

Several studies were carried out on reinforced concrete (RC) beams with openings to investigate the effect of opening size and shapes and mechanisms of crack initiation and propagation around these openings on the behavior of the beam [1,2,3,4]. RC beams with different opening sizes were experimentally studied [2]. The results showed that the opening existence decreases the capacity of the shear load, which is very dangerous because of the occurrence of brittle failure. Most of the cracks occurred around the shear-zone opening in the direction of the compression zone. Other cracks occurred at the moment zone and increased in length when the load was increased up the failure of the beam [2]. Deep beams with variable opening dimensions were studied [5]. The results showed that failure modes were significantly affected by the opening size and height ratios. The shear strength reduction was approximately 21% when the opening height (a) to beam depth (h) ratio increased from a/h = 0.3 to 0.4, and the shear strength reduction was approximately 51% when the opening height ratio increased from a/h = 0.3 to 0.5. There is a small effect on the behavior of the beam when the depth of the compression zone is more than the depth of compressive stress for the circular opening with a diameter of less than 44% of the beam depth [4]. 

In another study, the deflection increased by approximately 50%, and the load capacity decreased by approximately 50% because of the opening existing [6]. Openings with circular shapes were studied [3,7]. The effect of a small opening with a circular shape on the shear and flexural behavior and ultimate capacity of beams for normal and high strength concrete was presented [7]. The results show that the increase in opening diameter reduced the ultimate strength of the beam [7]. Deflection, strain, and cracks increased when the load increased up to failure. In addition, there is no significant effect on the behavior of the beams in the case of the circular opening with a diameter of 24% of the depth of the beam [3].

The roles of fiber-reinforced polymers (FRP) in the strengthening of structural elements were investigated by different research works [1,6,7,8,9,10]. Beams with a T-section and an opening on the web were studied [1]. Local carbon-fiber-reinforced polymer strengthening was used to increase the shear capacity of the post-weakening beam to obtain a ductile failure process. Two different dimensions of opening were considered—the first was 300 mm in height and 700 mm in length; the second was 280 mm in height and 800 mm in length. Results showed that the strengthening of RC beams with CFRP increases the load capacity and decreases the deflection of the beams. The results also showed that the height of the opening is more sensitive than its length, and this finding was attributed to the significant effect of the height of the neutral axis that increases the tension area and consequently increases the area of cracks.

The type of failure of RC beams with strengthened openings was studied [6]. There are two types of FRP failure, the first mode was FRP ruptures, and the second mode was debonding of FRP from the concrete surface [6]. To avoid the debonding of FRP layers, FRP wrapped around the opening, keeping 80 mm from the opening side at least. In this study, the load capacity increased by approximately 25% when strengthening the beam with FRP. In another experimental study, five large CFRP strengthened rectangular beams were damaged due to shear force [4]. The results show that strengthening and repair of damaged beams increase the stiffness of the beams and reduce deflection compared to the control beam. The load–deflection curve shows that the strengthened beams without steel stirrups have a very brittle failure to a sudden drop in the resisting load. After the application of strengthening, the ultimate shear capacity of the beam increased by approximately 100%. 

The efficiency of different strengthening techniques for drilled reinforced concrete beams was studied experimentally [11]. Diagonal bar and additional steel bar techniques were used around the opening before casting as internally reinforced, and externally bonded carbon-fiber-reinforced polymer sheets (CFRP) were used after casting. According to the results, it can be concluded that the strengthening technique type depends on the opening location.

Several techniques for strengthening RC beams containing openings were studied numerically [12]. The studied parameters also include the strengthening techniques and the opening locations. The FEM results were identical to the experimental results. The results proved that the suggested strengthening shapes improved the strength capacity, deflection, crack patterns, and the modes of failure of the beams.

The effect on the defects of externally bonded fiber-reinforced concrete was studied [13]. Defects in bonding cannot be avoided. Therefore, an experimental program including three-point bending and single shear pull-out tests was done on reinforced concrete beams with and without externally bonded FRP (EB-FRP). The effects of cracks with different widths and spacing were studied. The results show that even if more than 10% of the concrete was pulled off on the bond surface, the EB-FRP could retain its stable ductility and strength. Moreover, when the crack width is large, concrete cracking causes degradation of the bond-slip relationship. However, existing cracks have a minor influence on the load–deflection curves for strengthened RC beams.

Several works studied the behavior of concrete elements subjected to dynamic and blast loads [14,15,16,17,18]. Reinforced concrete structural elements are typically idealized as having perfectly elastoplastic member resistance and stepwise equivalent single degree of freedom (SDOF) transformation factors based on assumed elastic and plastic mode shapes. The use of idealized load-deformation characteristics, such as those recommended for blast resistant design by UFC, is entirely appropriate for preliminary analyses, r when more complex analyses are not warranted [17].

It was found that most of the experimental and numerical work that studied the opening in reinforced concrete beams focused on the openings that have sufficient steel reinforcement. The strengthening of the beams after construction or unreinforced openings was not sufficiently studied. In this paper, the subject of the analysis is a beam in which openings were defectively drilled after construction to simulate the actual behavior on-site; this is the main difference in this work. To perform that, an experimental program includes a control beam without opening and beams with unreinforced openings with and without bonded CFRP strengthening was performed. A numerical study was performed to investigate the effect of the opening on the behavior of the RC beams. The load–displacement relation and failure modes were used to analyze the behavior of the beams. A non-linear SDOF analysis was performed to investigate the approximate dynamic response of the beams, and a typical normal strength concrete under reinforced conditions was performed.

## 2. Experimental Study

The experimental work was designed to investigate the behavior of reinforced concrete beams with different geometry of openings in the constant moment region and the shear span. Intentionally the openings were designed without steel rebar to simulate the drilled opening after the beam construction. The effect of using carbon-fiber-reinforced polymers CFRP sheets in the strengthening of reinforced concrete beams containing openings was studied. From the economic and time-consuming point of view, only one specimen was tested for each configuration. It is common to use one RC specimen with or without strengthening elements for studying each parameter [19,20]. However, it is better to repeat each configuration to verify the findings and get conclusive evidence.

### 2.1. Experimental Program

**Figure 1:**
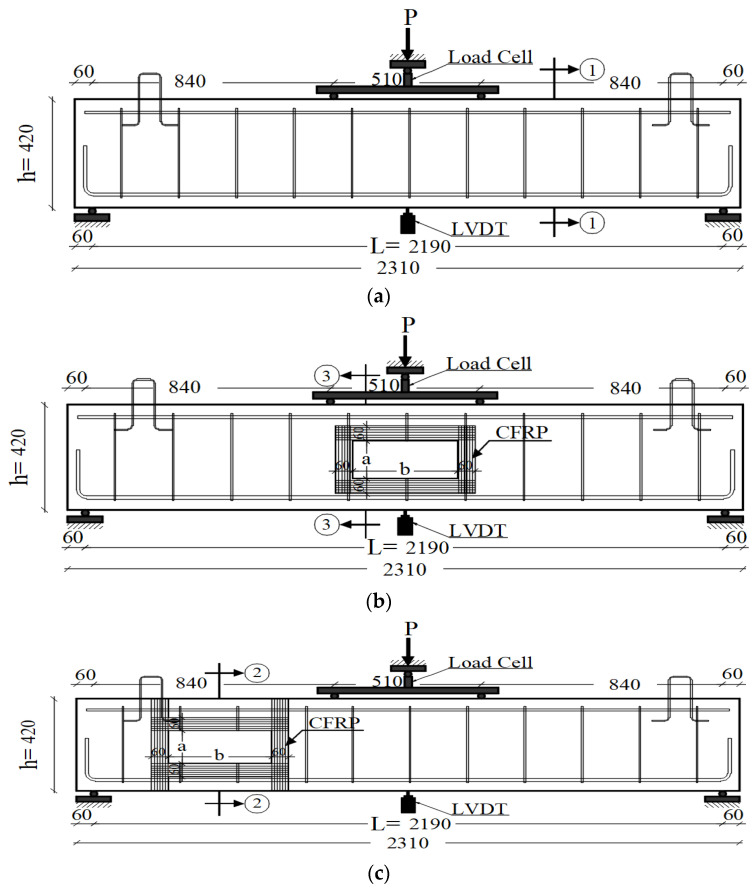

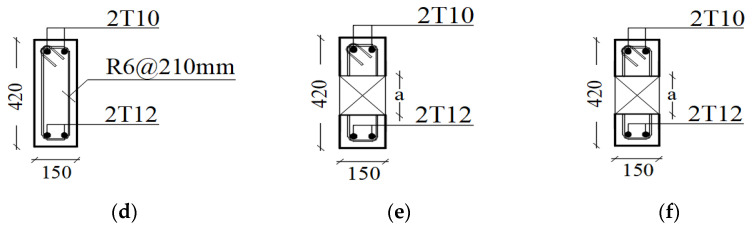
Experimental Sample Dimensions; (**a**) control beam; (**b**) opening in tension zone; (**c**) opening in the shear zone; (**d**) cross section ①-①; (**e**) cross section ②-②; (**f**) cross section ③-③ (dimensions are in mm).

Table 1 presents thirteen reinforced concrete beams that were fabricated and tested to investigate the behavior of reinforced concrete beams with relative opening dimensions, as shown in Figure 1a–d. The opening dimension are defined by the height (a) and the width (b). The beams had been loaded up to failure using a displacement control machine.

The experimental work was divided into two groups according to the location of the opening. In the first group, the openings were made in the maximum tension zone of the beams, while in the second group, the openings were made in one of the shear spans, as provided in Table 1. The first specimen is considered a reference beam for all other specimens, i.e., without opening. The other beams were divided into two groups; the first group represents the unstrengthened and strengthened beams with an opening in the constant moment region. The second group represents the unstrengthened and strengthened beams with an opening in the shear span. The strengthening was performed by using externally bonded CFRP sheets.

All the beams had the same dimensions, steel reinforcement, and material properties. The total length of the beams was 2310 mm with a 2190 mm span. All the beams had a rectangular cross-section of 420 mm in height and 150 mm in width. The main and the secondary steel reinforcement of the beams were two high tensile steel bars of 12 and 10 mm diameter, respectively. The shear reinforcement was 6 mm in the diameter stirrups of the mild steel bars with 210 mm fixed spaces, as shown in Figure 1. 

**Figure 2:**
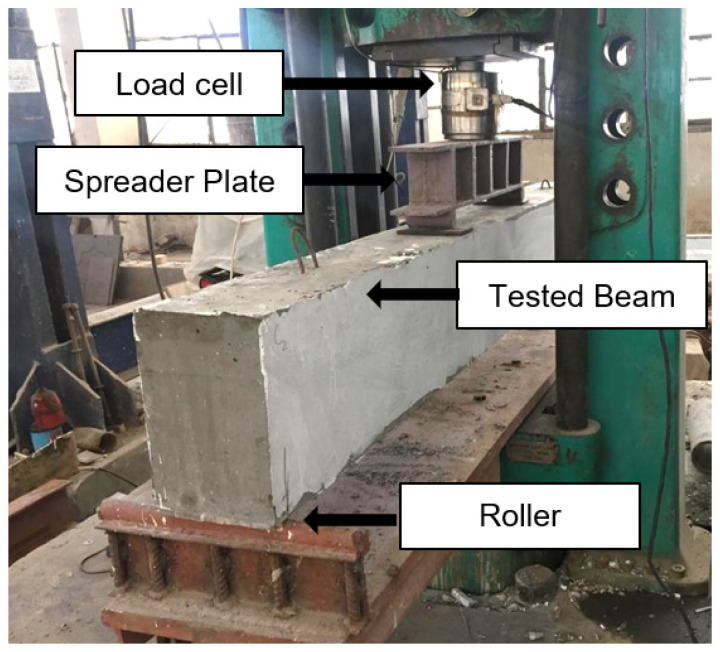
Sample Strengthening, Instrumentation and Loading.

These stirrups represent the minimum shear reinforcement of the control beam. One carbon-fiber-reinforced polymer (CFRP) layer was used to strengthen the beams, Figure 2. The beams were loaded with two-point loads at a distance of 510 mm apart, which represents the four-point bending case, Figure 2.

### 2.2. Material Properties

The quality control tests were carried out to determine the properties of the used materials (concrete, reinforcement, and CFRP). A compression test, according to Egyptian Standard Specification, ESS, 1658-6/2018, and BS EN 12390/2009, was performed for three concrete cubes sampled during the casting of each beam; all samples were tested after 28 days [21]. The average value of the compressive strength for tested samples is equal to 30.44 MPa with a standard deviation (STD) equal to 2.68 MPa. A tension test, according to Egyptian Standard Specification, ESS, 262-2/2015, and ISO 6935-2/2007, was performed for the steel-reinforcing bars. Three samples were tested for each diameter. The average test results for main steel and confinement bars are given in Table 2, with STD equal to 2.31 and 2.44 MPa for the main and confinement steel, respectively.

The CFRP fabric used to strengthen the specimens was unidirectional with nonstructural weaves in the transverse direction to hold the fabric together. According to the datasheet provided by the manufacturer, a dry fabric would have a tensile strength of 3.45 GPa, an elastic modulus of 230 GPa, and elongation at a break of 1.5% [22]. The fabric was laminated to the specimens with an epoxy resin having a tensile strength of 33.8 MPa and an ultimate elongation of 1.2%, according to Sikadur^®^-330 (Sika, Lyndhurst, NJ, USA) epoxy datasheet [23].

### 2.3. Test Procedure

By using a testing machine of 1000 kN capacity, the beams were tested until failure. Two rollers were used as supports for the beams. The load applied by the machine was in two points with a 510 mm distance between the loading points, as shown in Figure 2. The deflection was measured at the beam mid-span by using PY-2-F-010-S01M LVDT (linear vertical displacement transducers) (Gefran, Lombardia, Italy). All of the tested beams were painted with white color to simplify the observation of the cracks, and the formation and propagation of the crack were observed by the naked eye. A displacement-controlled hydraulic actuator was used to load the beam. A spreader beam was used to split the load into two equal loads. The spreader beam is rested on the beam by using two circular rods and two loading plates. Due to the asymmetry of the beam, the deflections of the two supports of the spreader beam are not equal. The end at the opening side always has higher deformation than the other end which cause the beam to rotate slightly. To overcome this problem, the connection between the actuator and the midpoint of the spreader beam was permitted to rotate to follow the deformation of the beam and maintain the equal load condition.

## 3. Numerical Study

In this section, the finite element modeling (FEM) methodology is described, including element type, material type, and contact details. The objective of this FEM work is to create an efficient numerical model that can be used to predict the response of the concrete beams with a drilled opening. To study the RC beams in the three-dimensional (3D) space, FEMs were developed. Each 3D model consisted of a solid block of reinforced concrete with or without opening and a thin shell layer of sheet strengthening. The concrete block was modeled by using 3D eight-node element SOLID65 [24]. This element showed an ability to simulate the behavior of reinforced concrete beams and FRP in several research works [25,26,27,28,29,30]. SOLID65 can handle both the concrete hardening and softening behaviors, and it can display the concrete cracks over the mesh. Eight-node SOLID180 elements were selected to simulate the rigid behavior of the bearing and loading plate, while BEAM 186 elements were used to create the longitudinal and transversal reinforcement. To model the strengthening sheet, the SHELL181 element was used, which can simulate the material and geometric nonlinearities. The contact element CONTA174 is used to represent contact and sliding between concrete, epoxy, and CFRP. In the case of pair-based contact, the target surface is defined by the 3-D target element type, TARGE170. The element has the same geometric characteristics as the solid or shell element face with which it is connected. Contact occurs when the element surface penetrates an associated target surface. 

**Figure 3:**
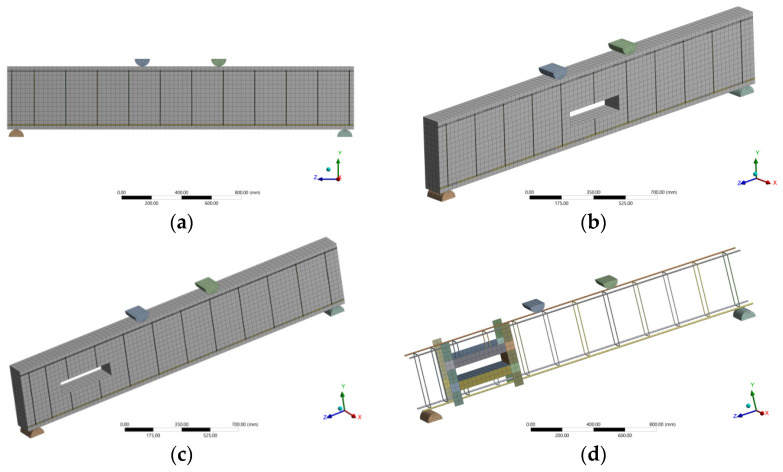
Finite Element Modeling. (**a**) control beam; (**b**) tension-zone opening; (**c**) shear-zone opening; (**d**) beam reinforcement.

Displacement control loading was conducted in the finite element model, where the displacement was applied to the top point of the loading plates, as shown in Figure 1, which closely simulates the experimental setup and test procedure. The finite element mesh of the control beam, the beam with tension-zone opening, and the beam with shear-zone opening are shown in Figure 3a–c, respectively. Cohesive zone modeling (CZM) was used to simulate the slippage behavior of the bond between the concrete and the strengthening sheet, see Figure 3d. The use of the CZM element requires defining failure displacement and failure stresses.

ANSYS concrete damage model was used to model the concrete beams. The stress–strain curve was used to simulate the hardening plasticity of concrete based on Equations (1) and (2) [31].

(1) $$f=\frac{E_{c}\;\varepsilon }{1+{\left(\frac{\varepsilon }{\varepsilon_{0}}\right)}^{2}}$$

(2) $$\varepsilon_{0}=\frac{2f{’}_{c}}{E_{c}}$$

where *f* is the concrete stress, *E_c_* is the concrete Young’s modulus, *ε* is the concrete strain, *ε*_0_ is the concrete failure compression strain, and *f′_c_* is the concrete compressive strength.

For the loading and bearing blocks, an elastic isotropic steel property was selected with Young’s modulus of 200 GPa and a Poisson ratio of 0.3. For the steel rebar, a bilinear elastoplastic model was used with Young’s modulus of 200 GPa and a Poisson ratio of 0.3. The yield strength was obtained from the coupon tests, and it was equal to 442 MPa, where 2000 MPa was used as a tangent modulus. The iterative, incremental load method is adopted in finite element analysis (FEA). The FEA covered the load versus deflection behavior, strain in concrete, CFRP, first crack load, number of cracks, the neutral axis height, and the capacity load. The main parameters of the finite element model are summarized in Table 3.

## 4. Experimental Results

In this section, the experimental results will be analyzed and discussed. The load–deflection curves of the beams with and without strengthening for all opening sizes will be discussed and compared to the control beam in detail.

### 4.1. Control Beam

**Figure 4:**
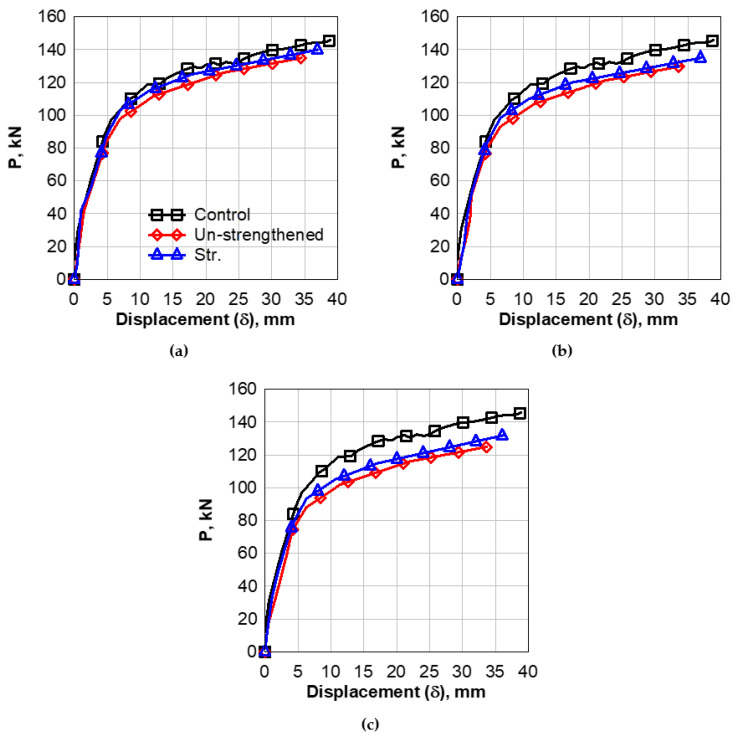
Load versus Mid-Span Deflection of Beams with Tension-Zone Opening. (**a**) opening 360 × 90 mm^2^; (**b**) opening 360 × 150 mm^2^; (**c**) opening 360 × 210 mm^2^.

The data in Figure 4 clearly shows that the load–deflection behavior exhibits a linear pattern up to a first crack load of 45 kN. After that, the relationship shows non-linear behavior up to failure load. The maximum load of the control beam is 145 kN, and the mid-span deflection at first crack load is 3 mm, while the max deflection is 40 mm.

**Figure 5:**
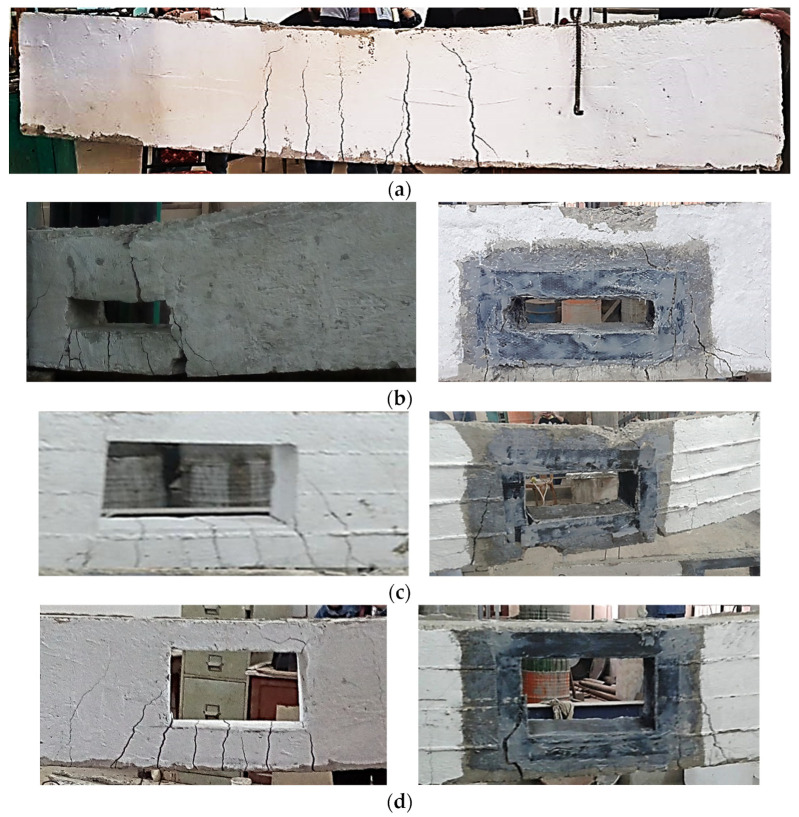
Failure Modes of Samples with Tension-Zone Opening. (**a**)control Beam; (**b**) opening 90 × 360 mm^2^; (**c**) opening 150 × 360 mm^2^; (**d**) opening 210 × 360 mm^2^.

The tension cracks propagated from the mid-bottom section of the beam until it reached nearly the whole depth, is illustrated in Figure 5. Irreversible plastic deformation was observed in the beam due to the bottom ductile reinforcement.

### 4.2. The Behavior of Drilled Beams in Tension-Zone

In this section, the results of drilled beams in the tension zone will be discussed. The load–displacement analysis, including the first crack load, the failure load, and the maximum displacement, will be analyzed, see Figure 4. The failure mode of the sample and the effect of opening size and opening strengthening were studied and are illustrated in Figure 5.

#### 4.2.1. Load–Displacement Behavior

For the case of 90 × 360 mm^2^ opening without strengthening, the data in Figure 4a clearly shows that the load–deflection behavior exhibited a linear pattern up to a load of 41.5 kN, i.e., the first crack load. After that, the relationship exhibited non-linear behavior up to failure load. The max load of this beam was 135 kN, and the mid-span deflection at first crack load was 1.5 mm, while the max deflection was 34.5 mm. 

As seen in Figure 4a, it can be detected that the tension-zone opening caused a decrease in the value of the first cracking load from 45 kN for the undrilled control beam to 41.5 kN for the drilled beam with a percentage decrease of 8%. Moreover, the value of the maximum load decreases from 145 kN to 135 kN, with a percentage decrease of 7%. The CFRP strengthening caused an increase in the value of the first cracking load from 41.5 kN for the drilled beam to 42 kN for the strengthened beam; the percentage increase was 1.2%. Moreover, the value of the maximum load increased from 135 kN for the drilled beam to 140 kN for the strengthened beam; the percentage increase was 4%.

Similar behavior was observed for the drilled beam with an opening of 150 × 360 mm^2^, as seen in Figure 4b. The load–deflection behavior exhibited a linear pattern up to a load of 37 kN, which was considered the first crack load. The maximum load of this beam was 130 kN, and the mid-span deflection at first crack load was 2 mm, while the maximum deflection was 34 mm. Comparing the results to the control beam could show the effect of the opening on the behavior of the RC beam. The existence of the opening caused a decrease in the value of the first cracking load from 45 kN for the control beam to 37 kN for the drilled beam, where the decrease percentage was approximately 18%. Moreover, the value of the maximum load decreased from 145 kN for the control beam to 130 kN for drilled beam; the percentage reduction was approximately 11%. For the case of the beam with CFRP strengthening, it can be detected that the strengthening of the beam caused an increase in the value of the first cracking load from 37 kN for the drilled beam to 38 kN for the strengthened beam, the percentage of enhancement was 3%. Moreover, the value of the maximum load increased from 130 kN for the drilled beam to 135 kN for the strengthened drilled beam with an enhancement percentage of 4%.

The behavior of the beams with an opening of 210 × 360 mm^2^ is shown in Figure 4c. It can be observed that the load–deflection behavior exhibited a linear pattern until a first crack load of 31.5 kN. The non-linear behavior started after that load up to a failure load of 125 kN for that beam. The mid-span deflection at first crack load was 1.5 mm, while the maximum deflection was 33.75 mm. It can be detected that the opening of the RC beam, caused a remarkable decrease in the value of the first cracking load from 45 kN for the control beam to 31.5 kN for drilled beam, with a 30% percentage of decrease. In addition, the value of the maximum load decreased from 145 kN for the control beam to 125 kN for the drilled beam, with a reduction percentage of 14%. Furthermore, strengthening the opening with the FRP sheets caused an increase in the value of the first cracking load from 31.5 kN for the drilled beam to 32 kN for the strengthened drilled beam. The value of maximum load increases from 125 kN for the drilled beam to 132 kN for the strengthened drilled beam with a percent of 6%.

#### 4.2.2. Failure Analysis of Tension-Zone Drilled Beams

Figure 5 shows the failure of all the beams, for all unstrengthened beams, the tension cracks started at the bottom concrete fiber of the beam and spread until the part underneath the opening fully cracked. The crack appeared at the top part of the opening by increasing the load, starting from the opening top boundary. For samples with opening heights of 90 and 150 mm, the final failure happened due to the tension rebar rupture, Figure 5b,c. For the sample with an opening height of 210 mm, the remaining compression zone was not able to carry the compression force, and crushing failure appeared at the top of the beam, Figure 5b.

For the strengthened sample with a 90 mm opening height, tension cracks appeared on the bottom fiber of the beam outside the reinforced zone and spread toward the top of the beam, Figure 5b. Crushing at the compression zone was observed at the top of the beam, and no delamination in the FRP layers was observed. The CFRP sheets prevented the tension failure of the bottom steel, which led to an increase in the load and initiated the crushing compression failure. For the beams with 150 and 210 mm opening heights, delamination failure was observed at the bottom side of the opening, combined with tension and compression cracks in the concrete, as shown in Figure 5c,d.

#### 4.2.3. Effect of Strengthening and Opening Size on Beams Behaviors

**Figure 6:**
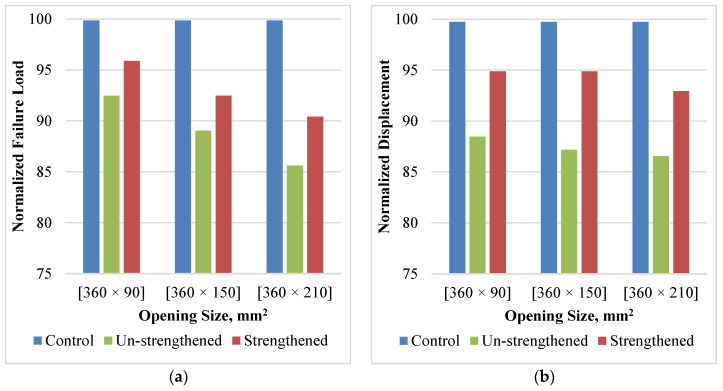
Effect of Strengthening and Tension Zone Opening Size. (**a**) failure load; (**b**) failure displacement.

Figure 6 shows the normalized failure load and displacement results of all the tension-zone opening cases. The failure load was normalized by finding the percent between the failure load of each case and the failure load of the control beam case. For the case of unstrengthened openings, the existence of openings led to a decrease in the maximum load to 92, 89, and 86% of the control load for the 360 × 90, 360 × 150, 360 × 210 mm^2^ openings, respectively, Figure 6a. As expected, by increasing the opening height, the maximum failure load decreased; however, the tension-zone openings do not have a significant effect on decreasing the strength. As a result, no significant strength enhancement was observed after strengthening the drilled beams with the FRP sheets; the enhancement ratio ranged from 3.0 to 5.0%. Although the strengthening enhancement was marginal compared to the strength of the control beam, the ratio between the strength gained due to the strengthening and the strength lost due to the opening was between 31 and 46%. 

The failure displacement was normalized by finding the percent between the failure displacement of each case and the failure displacement of the control beam case. The failure displacements of the three cases of the drilled beams were 88, 87, and 87% of the maximum control beam displacement, Figure 6b. This leads to a decrease in ductility, which has a negative effect on the performance of the beam under dynamic loads such as blast and seismic loads. The enhancements of the maximum displacement were 6, 8, and 6% for the beam with openings 360 × 90, 360 × 150, and 360 × 210 mm^2^, respectively.

### 4.3. The Behavior of Drilled Beams in the Shear Zone

**Figure 7:**
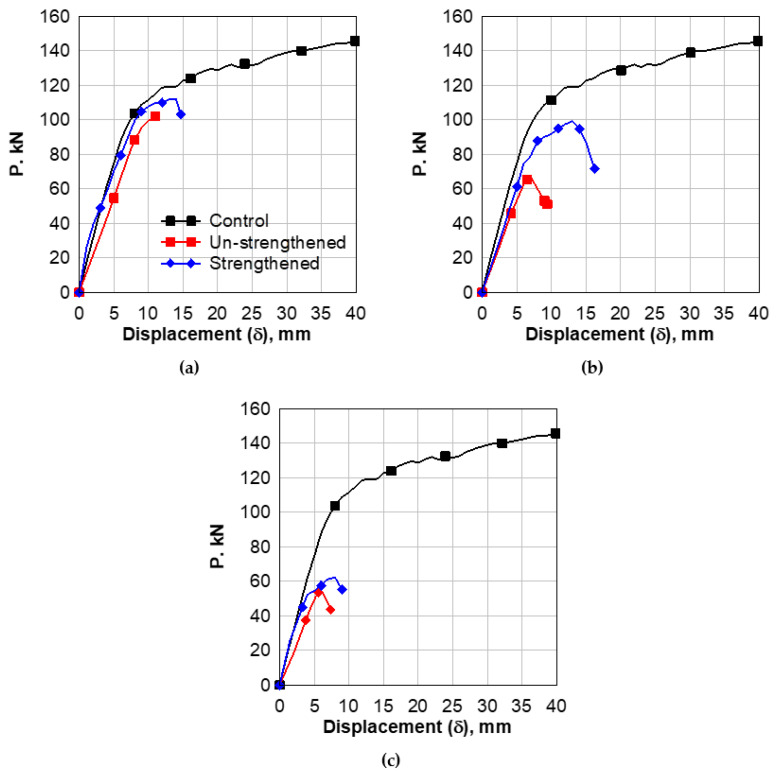
Load versus Mid-Span Deflection of Beams with Shear-Zone Opening. (**a**) opening 360 × 90 mm^2^; (**b**) opening 360 × 150 mm^2^; (**c**) opening 360 × 210 mm^2^.

**Figure 8:**
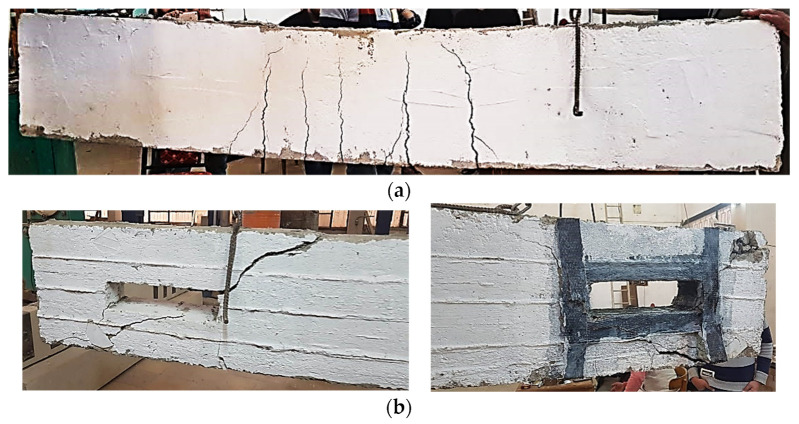

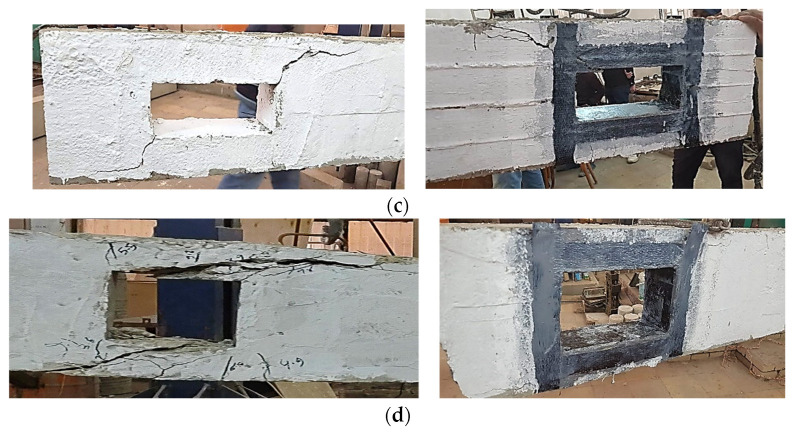
Failure Model of Samples with Shear-Zone Openings. (**a**) control beam; (**b**) opening 90 × 360 mm^2^; (**c**) opening 150 × 360 mm^2^; (**d**) opening 210 × 360 mm^2^.

**Figure 9:**
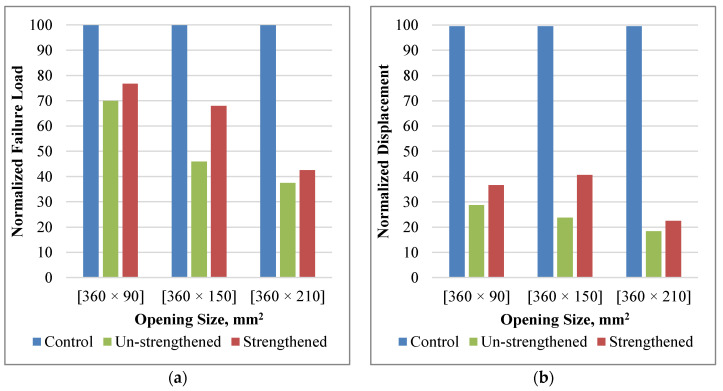
Effect of Strengthening and Shear Zone Opening Size. (**a**) failure load; (**b**) failure displacement.

This section will discuss the results of drilled beams in the shear zone, as shown in Figure 7, Figure 8 and Figure 9. The load–displacement analysis is analyzed, including the first crack load, the failure load, and the maximum displacement. The failure mode of the sample and the effect of opening size and opening strengthening will be a focus.

#### 4.3.1. Load Deflection Behavior

The load–deflection behavior of beams with an opening of 90 × 360 mm^2^ in the shear zone is shown in Figure 7a. This sample exhibited a linear load–deflection pattern up to load = 32 kN, which can be considered the first crack load. This relationship showed non-linear behavior up to the failure at a maximum load of 102 kN. The mid-span deflection at first crack-load was 3 mm, while the maximum deflection was 11.5 mm. The existence of an opening caused a remarkable decrease in the value of the first cracking load from 45 kN for the control beam to 32 kN for a drilled beam, with a percentage decrease of 28.89%. The value of the maximum load decreased from 145 kN for the control beam to 102 kN for a drilled beam; the percentage decrease was 29.66%. The results of the strengthened beam are found in Figure 7a; the strengthening caused a remarkable increase in the value of the first cracking load from 32 kN for the drilled beam to 39.5 kN for the strengthened drilled beam with a percent of 18.99%. Moreover, the value of maximum load increases from 102 kN for the drilled beam to 112 kN for the strengthened drilled beam.

Figure 7b shows the load–deflection curve of the case of opening 150 × 360 mm^2^. The first crack load was 23.2 kN, and the maximum load was 67 kN. The mid-span deflection at the first crack load was 2.2 mm, and the maximum deflection was 7 mm. The existence of the opening caused a remarkable decrease in the value of the first cracking, with a percent of 48.44%, and the maximum load decreased by 53.79%. The CFRP strengthening causes a remarkable increase in the value of the first cracking load, with a percent of 34.45%, and the maximum load increased by 32.46% over the unstrengthened beam.

Figure 7c shows the load–deflection curve of the case of opening 210 × 360 mm^2^. The first crack load was 18.97 kN. Afterward, the samples showed a non-linear behavior up to a failure load of 54.77 kN. The mid-span deflection at first crack load was 2.1 mm, while the max deflection was 6 mm. The opening causes a remarkable decrease in the value of the first cracking load by 57.84% and a 62.23% decrease for the maximum load under the control beam. The strengthening with CFRP causes a remarkable enhancement in the value of the first cracking load by approximately 26.19%, and the value of maximum load increased from 11.95% over the unstrengthened drilled beam.

#### 4.3.2. Failure Analysis of Tension-Zone Drilled Beams

Figure 8 shows the failure of all the beams; for all unstrengthened beams, the shear cracks at the top-left and bottom-right corners of the beam spread with 45° toward the top and bottom of the beam until the cracks fully opened. For the strengthened sample with a 360 × 90 mm^2^ opening, tension cracks appeared on the bottom fiber of the beam outside the reinforced zone and spread toward the top of the beam. Crushing in the compression zone was observed at the top of the beam, and no delamination in the FRP layers was observed. For the beam with 360 × 150, 360 × 210 mm^2^ openings, delamination failure was observed in the bottom side of the opening combined with tension and compression cracks in the concrete.

#### 4.3.3. Effect of Strengthening and Opening Size on Beams Behaviors

Figure 9 compares all the tested beams together; the failure load was normalized by finding the percent between the failure load of each case and the failure load of the control beam case. The existence of opening led to a significant decrease in the maximum load of 70, 46, and 39% of the control beam load for the unstrengthened beams with 360 × 90, 360 × 150, and 360 × 210 mm^2^, respectively, Figure 9a. By increasing the opening height, the maximum failure load decreases due to the reduction of the shear section. Strengthening with the FRP sheets enhanced the failure load of the drilled beams by 7, 22, and 5%, where the maximum enhancement was for the beam with a 360 × 150 mm^2^ opening. 

The failure displacement was normalized by finding the percent between the failure displacement of each case and the failure displacement of the control beam case, Figure 9b. Drilling the opening decreased the failure displacement significantly, which negatively affects the response of the beams under dynamic loads such as the blast and seismic loads. This enhanced the failure displacement by a maximum percentage of 17% for the beam with a 360 × 150 mm^2^ opening.

## 5. Energy Absorption of the Beams

**Figure 10:**
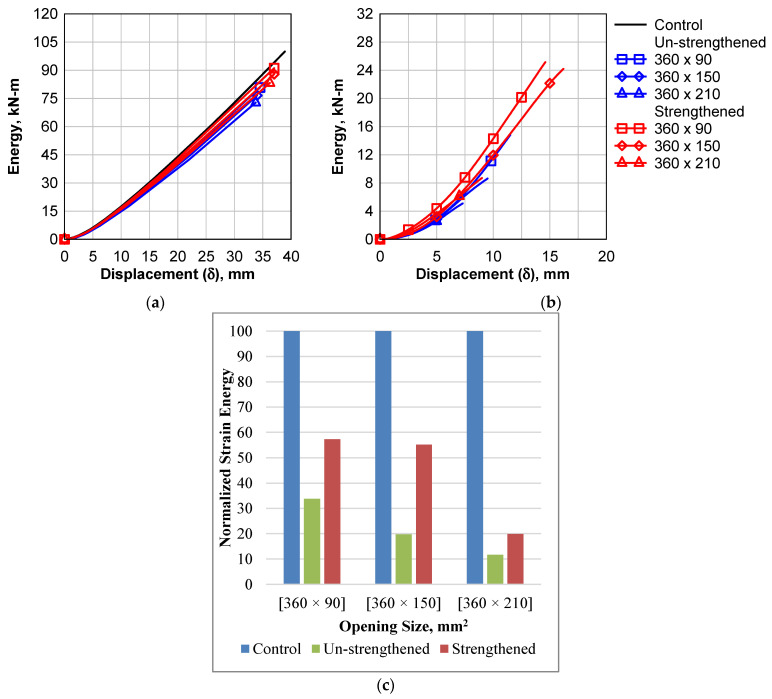
Energy Absorption. (**a**) tension zone opening samples; (**b**) shear zone opening samples; (**c**) normalized maximum energy of shear zone opening samples.

The ability of the structural system to absorb large amounts of strain energy before the final collapse is an indication of the efficiency of this system against the dynamic loads. The amount of strain energy (SE) absorbed was calculated for each beam was calculated, as shown in Figure 10. It can be found that the tension-zone openings do not show a significant reduction in the absorbed energy, see Figure 10a. The maximum absorbed energy of the control beam was 4.67 kN-m. The maximum reduction due to the tension opening was approximately 28% for the beam with an opening of 360 × 210 mm^2^. The strengthening of this beam enhanced the energy absorption behavior of the beam, and the reduction ratio became 17%. The effect of shear opening on the energy absorption was a sever effect, see Figure 10b,c. In Figure 10c, The strain energy was normalized by finding the percent between the strain energy of each case and the strain energy of the control beam case. The sample with 360 × 210 mm^2^ lost approximately 95% of the control beam energy absorption capacity. Although the strengthening of this beam enhanced the energy absorption behavior of the beam and the reduction ratio became 92%, it is not recommended to use the opening in the shear zone for concrete beam subjected to dynamic loads.

## 6. Numerical Modeling Results

In this section, the finite element model results will be discussed. The comparisons between the load–displacement curves of the FE model and experimental tests will be described. The objective of this section is to develop a model that can simulate the beams with openings accurately, which can be used in the future to investigate different parameters.

### 6.1. Control Beam

**Figure 11:**
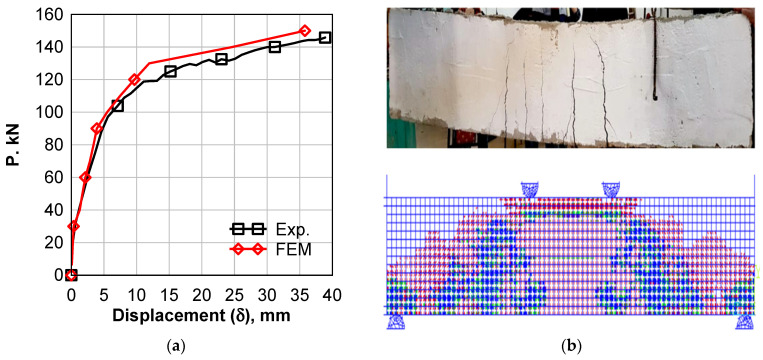
Comparison between Experimental and Numerical Results of Control Beam. (**a**) load against mid-span deflection; (**b**) concrete cracks.

Figure 11a shows a comparison between the load–deflection curves of experimental tests and numerical simulation of the control beam. Both the numerical and experimental results exhibit linear behavior up to the first crack load, which was 45 kN and 40 kN for experimental and numerical, respectively, with a 33% difference. After that, the relationship exhibits non-linear behavior up to failure load. The maximum load was 145 kN for the experimental and 150 kN for the numerical results of the control beam, with a 4% difference. The mid-span deflection at maximum load is 40 mm and 35.8 for experimental and numerical, respectively, with a 10.5% difference. The numerical vs. experimental crack pattern is shown in Figure 11b; it can be found that the tension cracks collected mainly at the constant bending moment zone. A combination of shear and tension cracks was found at the constant shear area between the loading point and supports of the beam. It can be concluded that the numerical model was able to predict both the maximum load and failure pattern of the control beam.

### 6.2. Comparison of Drilled Beams Results

**Figure 12:**
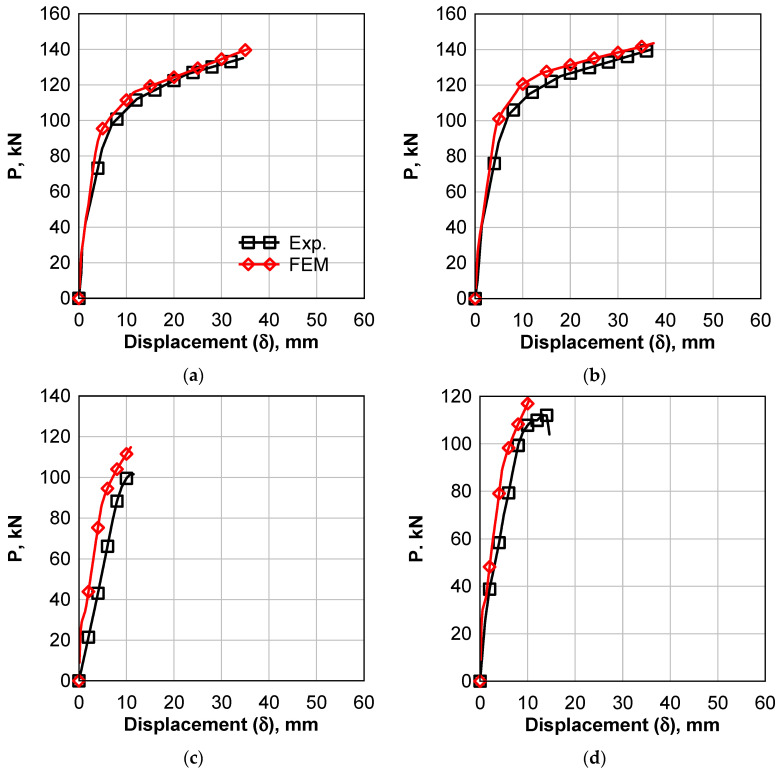
Experimental versus Numerical Load Displacement Curves of Beams with 360 × 90 mm^2^ Opening. (**a**) unstrengthened tension; (**b**) strengthened tension; (**c**) unstrengthened shear; (**d**) strengthened shear.

Figure 12a,b show a comparison between the numerical and experimental results for the drilled beam with a 360 × 90 mm^2^ opening in the tension zone. The load–deflection behavior exhibited a linear pattern up to the first crack load, which were 41.5 kN and 22.5 kN for experimental and numerical results, respectively, with a 46% difference for unstrengthened beams. The same difference was observed between the experimental and numerical first crack load of the strengthened beams, Figure 12b. After that, the relationship showed a non-linear behavior up to failure load. For the unstrengthened beams, the maximum loads were 135 kN and 145 kN for experimental and numerical, respectively, with a 7.4% difference. For the strengthened beam, the experimental and numerical loads were 140 kN and 150 kN, respectively, with a 7.14% difference. The mid-span deflection at maximum load was 34.5 mm and 29.42 for the unstrengthened beams experimental and numerical results, respectively, and 37 mm and 25.11 for strengthened beams. For the drilled beams with a 90 × 360 mm^2^ shear-zone opening, the load–deflection curves are shown in Figure 12c,d. The first crack loads of the unstrengthened beams were 32 kN and 24 kN for experimental and numerical, respectively, with a 25% difference. The first crack-load difference in the case of the strengthened beam was 37%. The maximum loads of the unstrengthened beams were 102 kN and 115 kN for experimental and numerical results, respectively, with a 13% difference; however, the difference was 6% for the strengthened beams. It can be concluded that the numerical model can predict the maximum load accurately. The prediction of maximum displacement for the case of the shear opening case was better than the tension-zone opening case.

**Figure 13:**
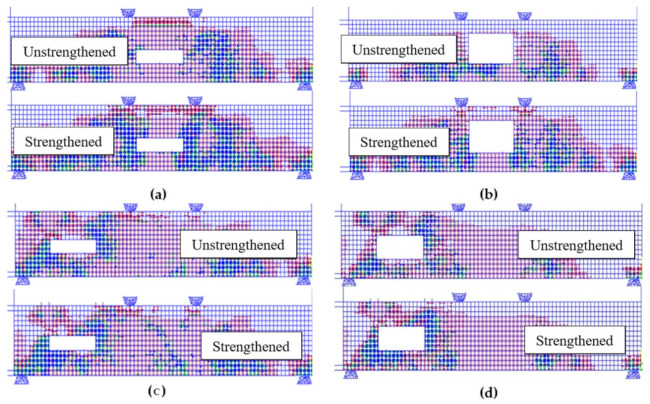
Numerical Crack Patterns. (**a**) tension 360 × 90 mm^2^; (**b**) tension 360 × 210 mm^2^; (**c**) shear 360 × 90 mm^2^; (**d**) shear 360 × 210 mm^2^.

Figure 13 shows the numerical concrete cracking patterns for both 360 × 90 and 160 × 210 mm^2^ cases with and without strengthening. The cracks in the tension-zone opening samples shown in Figure 13a,b are mainly tension cracks at the constant moment zone and shear cracks at the constant shear-zone. Some compression-crushing crack appeared in the unstrengthened sample near the top fiber at the mid-span of the concrete beam for the sample with a 360 × 90 opening. Shear cracks also were observed at the corners of the openings, although they exist in the tension zone. A reduction in the density of cracks can be observed after the strengthening as the FRP sheet decreases the stress in the concrete. The cracks of the tension-zone opening samples are shown in Figure 13a,b. The cracks are mainly shear cracks at the top-right and bottom-left corners of the opening. At the mid-span, the shear cracks completely disappeared.

## 7. Conclusions

In this paper, an experimental and numerical investigation was conducted to study the effect of a drilled opening on the performance of a reinforced concrete beam. For simplicity, instead of the concrete drilling after construction, reinforced concrete samples with rectangular openings in the shear and tension zones were prepared. Based on the experimental and numerical results, the following conclusions can be drawn:

- The tension-zone opening did not significantly decrease the strength of the beam. The maximum failure load reduction was 14% for the 360 × 120 mm^2^ opening compared to the undrilled control beam.
- Although the strengthening enhancement was marginal compared to the control beam strength, the strength of the strengthening beams increased by approximately 31 to 46%, which represents the efficiency of the strengthening.
- Sever strength and ductility reductions were observed in the case of the shear-zone drilled openings due to the absence of shear reinforcement. The strength and ductility reductions due to the openings ranged from 30 to 62%. The CFRP strengthening enhanced both the strength and displacement by 5–22% and 4–17%, respectively.
- The efficiency of the strengthening in recovering the lost strength was 8–40%. Therefore, it is not recommended to drill any opening at the shear zone as strength loss can reach 57% even with the strengthening.
- The opening also badly affected the failure displacement and absorbed strain energy by a reduction percentage of 63–81% and 66–88%, respectively.
- The developed finite element modeling was able to closely predict the structural behavior of the RC beams with openings.
- A preliminary dynamic blast analysis was performed. It was found that although the strengthening enhanced the blast response of the drilled beam, it is not recommended to drill any openings in the blast resistance structures.

## Figures and Tables

**Table 1:** Opening Dimensions for Group No. 1 and Group No. 2.

Beam No.	Group	Location of the Opening	Opening Dimensions (a × b), mm^2^	Relative Dimensions, (a/b)	CFRP Strengthening
B1	Reference beam	~	~	~	No
B2	1	In the constant moment region	90 × 360	0.21 (90/420)	No
B3			90 × 360	0.21	Yes
B4			150 × 360	0.36	No
B5			150 × 360	0.36	Yes
B6			210 × 360	0.50	No
B7			210 × 360	0.50	Yes
B8	2	In the shear span	90 × 360	0.21 (90/420)	No
B9			90 × 360	0.21	Yes
B10			150 × 360	0.36	No
B11			150 × 360	0.36	Yes
B12			210 × 360	0.50	No
B13			210 × 360	0.50	Yes

**Table 2:** Tension test results for main steel and confinement bars, according to the Egyptian Standard Specification, ESS, 262-2/2015, and ISO 6935-2/2007.

Steel/Property	Yield Strength, MPa	Tensile Strength, MPa	% Maximum of Elongation
Main steel, Grade 440	440	600	20
Confinement steel, Grade 300	300	450	25

**Table 3:** Details of the Finite Element Model.

Component	Element Type	Material Model
Concrete	SOLID 65	Concrete Model
Rebar	BEAM 186	Bilinear Isotropic Hardening
FRP Strips	SHELL 181	Elastic Orthotropic
Contact	CONTA 174 and TARGE 170	CZM Material
Loading and Bearing Plates	SOLID 180	Elastic Isotropic
